# Lateral pillar is the key in supporting pre-collapse osteonecrosis of the femoral head: a finite element model analysis of propensity-score matched cohorts

**DOI:** 10.1186/s13018-021-02875-8

**Published:** 2021-12-20

**Authors:** Ji Hoon Bahk, Woo-Lam Jo, Seung-Chan Kim, Soon-Yong Kwon, Young Wook Lim

**Affiliations:** 1 Department of Orthopedic Surgery, Seoul St. Mary’s Hospital, School of Medicine, The Catholic University of Korea, 222, Banpodae-ro, Seocho-gu, Seoul, 06591 South Korea; 2 Department of Orthopedic Surgery, Eunpyeong St. Mary’s Hospital, School of Medicine, The Catholic University of Korea, Seoul, South Korea

**Keywords:** Finite element analysis, Osteonecrosis of the femoral head, Lateral pillar, Femoral head collapse, Propensity-matched score

## Abstract

**Background:**

This study was designed as a cohort study using propensity-score matching to age, gender, and body mass index (BMI) for finite element model (FEM) analysis from pre-collapse CT images of collapsed and non-collapsed hips. Through FEM analysis, a global graphical output around the hip joint can provide simple impression of stress distribution: concentration or dispersion.

**Methods:**

A total of 32 hips with ARCO stage 2 or 3 ONFH who were on follow up for over a one-year period were retrospectively reviewed. 16 hips with no interval progression of collapse were set as the study group, then 16 hips with progression of collapse which required arthroplasty were set as the control group using propensity-score matching. FEM was generated through Mechanical Finder for each patient, then 4500 N of load was applied to 1000 mm^2^ area at the top of iliac crest to analyze the models in terms of equivalents for yield stress.

**Results:**

Age, sex, and BMI had no significant differences between the two groups, while location (*p* = 0.015) was lateral, and size (*p* = 0.015) was significantly greater in the collapsed group. Non-collapsed hips mostly exhibited stress dispersion allocated to medial and lateral pillars, while collapsed hips exhibited stress concentration focused on the lateral pillar and the primary compression trabecula. (*p* = 0.001).

**Conclusion:**

Through FEM analysis, stress concentration to the lateral pillar and the primary compression trabeculae can be used to predict future collapse in ONFH with high probability. Results provide a simple and intuitive, yet valuable information to aid surgeons. Therefore, especially for young patients, holding out the lateral pillar through joint preserving procedures might be the key in preventing further collapse.

## Background

The natural history of osteonecrosis of the femoral heads (ONFH) is still an area of uncertainty in part, since some patients with early stages of ONFH might undergo collapse in time while others do not exhibit any progression throughout the follow up period. Therefore, the matter of utmost concern to surgeons has been the prediction of collapse progression of the femoral head [1]. In an effort, various staging systems based on necrotic size, location, and presence of subchondral fractures or collapse have been widely used in practice. Among them, Ficat and Alert classification [2], University of Pennsylvania system [3], the Japanese Investigation Committee (JIC) classification [4], and ARCO (Association Research Circulation Osseous) international classification of osteonecrosis have been widely used in order to make the ultimate treatment choice: surgery or observation. Subsequently, Kerboul combined necrotic angle was proposed to predict collapse using sum of necrotic angles in MRI images [5]. Another indicator of collapse suggested by another latest study is maximum area in coronal position (MAC) of initial bone resorption [6].

More recently, since the introduction and application of finite element model (FEM) analysis on ONFH, novel studies have been reported in advanced attempt to reveal better indicators [7]. Through FEM study, Utsunomiya et al*.* concluded that lateral boundaries of the necrotic lesion lead to subchondral fractures and collapse [8]. Additionally, in FEM interpretation via peak von Mises stress, Wen et al*.* emphasized the significance of the lateral pillar in progression of the disease [9, 10], and Li et al. suggested maximum level of stress on weight-bearing surfaces as a new biomechanical marker for the prediction of collapse [11].

We designed a retrospective cohort study using propensity-score matching to age, gender, and BMI for FEM analysis from pre-collapse CT scan images of collapsed and non-collapsed hips. ARCO staging, size, and location were primarily compared between the two cohorts. Furthermore, FEM was generated to determine how the stress is distributed at the femoral head, especially in the lateral pillar and the primary compression trabeculae. Through FEM analysis, rather than locally focusing on quantification, a global graphical output around the hip joint was obtained which gives us a simple impression of the stress distribution: concentration or dispersion.

## Methods

This study was approved prior to initiation by the Institutional Review Board. Informed consent was waived by the board. In total, 32 hips in 32 distinct patients with ARCO stage 2 or 3 ONFH which were diagnosed with both plain radiographs and MRIs who were on follow up for over than one-year period between January 2016 and December 2018 were retrospectively enrolled in this study. In total, 16 hips with no interval progression of collapse were set in the study group (group A), then in turn, 16 hips with progression of collapse which eventually required arthroplasty were set as the control group (group B) by using propensity-score matching to age, sex, and body mass index (BMI).

### Finite element model generation

For analysis, three-dimensional FEM of each patient were generated using Mechanical Finder version 10.0 (Research Center for Computational Mechanics, Tokyo, Japan) and digital imaging and communication in medicine (DICOM) images which were obtained from routine initial pelvic bone CT (120 kVp, 1.0 mm slice thickness, SOMATOM Definition, Siemens Medical Solutions, Forchheim, Germany). Extracted DICOM images were imported into Mechanical Finder, then region of interests (ROI) were initially extracted using computational methods to firstly select coarse areas over arbitrary Hounsfield unit thresholds. Then additionally, fine ROI boundaries were selected by meticulous manual identification to identify more accurate necrotic areas for the FEM. Subsequently, ANSYS version 19.2 (ANSYS, Inc., Canonsburg, Pennsylvania, USA) was loaded to Mechanical Finder for mesh generation.

For outer surface of the cortical bone, 0.5 mm thickness iso-surface external mesh was automatically generated while for the trabecular bone, internal mesh was generated using 1 mm-sized, 10-node tetrahedral elements. Then stepwise material property was configured with inhomogeneous bone material settings using Keyak (1998) preset of conversion equation [12] for Young’s modulus, yield stress value, critical stress value, Poisson’s ratio, and strain relaxation coefficients. Accordingly, Poison’s ratio was set to 0.4 and lower limit of Young’s modulus was set as 14.71 MPa for the pelvic bone and the proximal femur. For articular cartilage, it was homogenously set to 0.4 for Poisson’s ratio, and 10.35 MPa for Young’s Modulus [8, 12]. Additionally, distribution of Drucker–Prager was utilized for yield criterion.

**Fig. 1:**
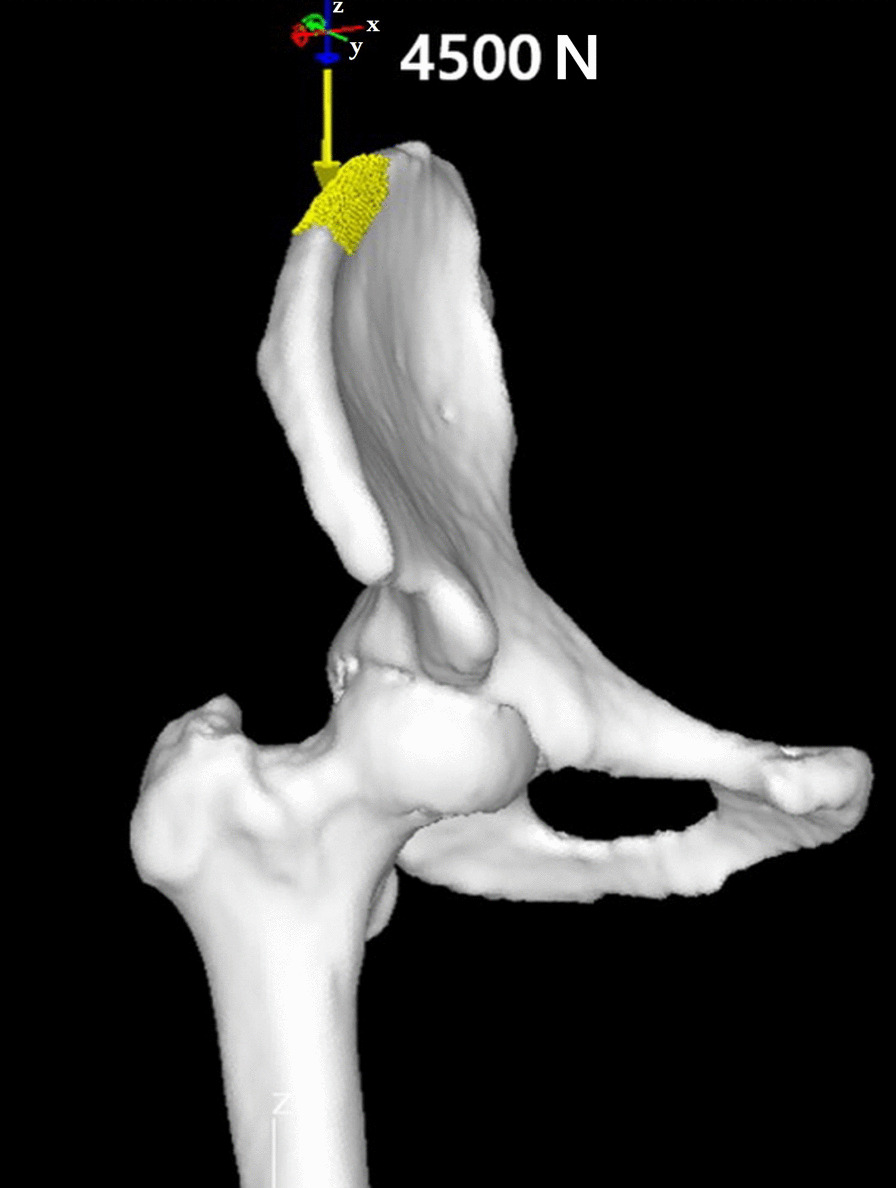
Virtual load application to FEM. Load of 4500 N was applied to arbitrary 1000 mm^2^ area at the top of iliac crest with vector parallel to the vertical axis of a body (arrow)

**Fig. 2:**
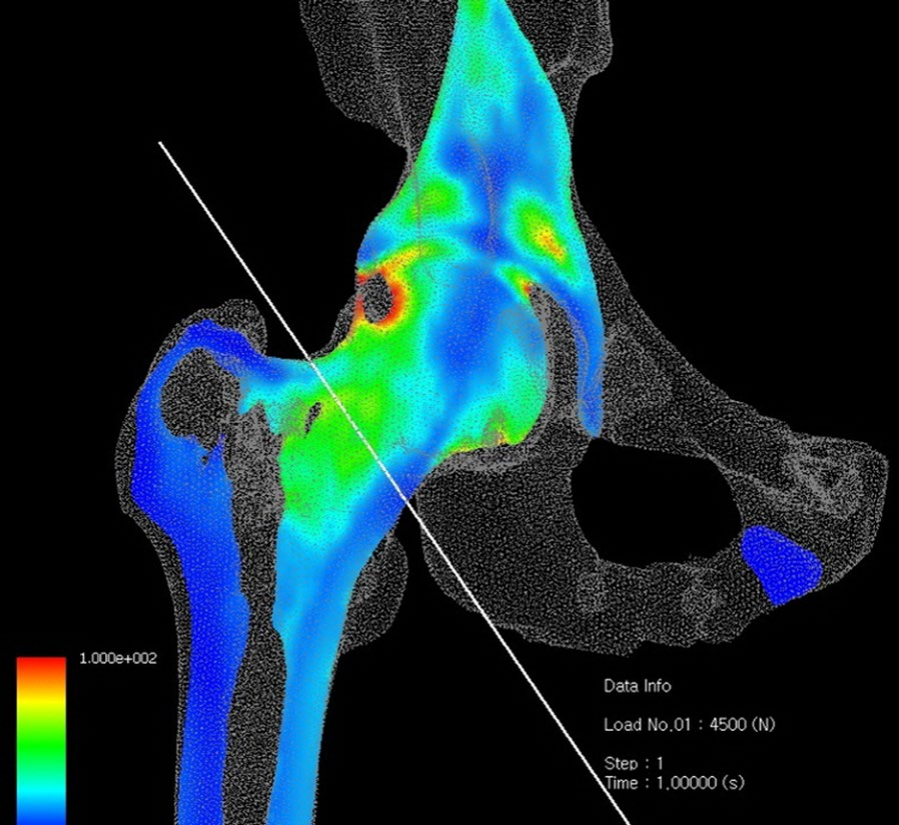
Final graphical output image after FEM analysis: resulting stress distribution across the hip joint, through the proximal femur. Stress dispersion through medial and lateral cortices is noted in this case

After completion of FEM generation, stress load of 4500 N was added to manually pointed 1000 mm^2^ area at the top of ipsilateral iliac crest, with the force vector parallel to the vertical axis of the body (Fig. 1). The force was fully restrained at proximal one-third of the femoral shaft, further distal to the subtrochanteric area. Ultimately, final analysis was performed to yield qualitative and quantitative results of the stress distribution around the hip joint, which includes three-dimensional output figures (Fig. 2).

### Study outcomes

Changes in necrotic areas were reassessed using CT images at follow up and was again classified using ARCO international classification of osteonecrosis. Additionally, the location of center of necrosis was assessed in accordance to trisections of femoral head on coronal plane [13]: medial, central, or lateral. The size of bone affected by necrosis was measured by the percent area (extent) of the necrotic portion using proportional expression which includes the longest mediolateral and anteroposterior length of necrotic lesion and the largest mediolateral and anteroposterior diameter of the femoral head [14].

**Fig. 3:**
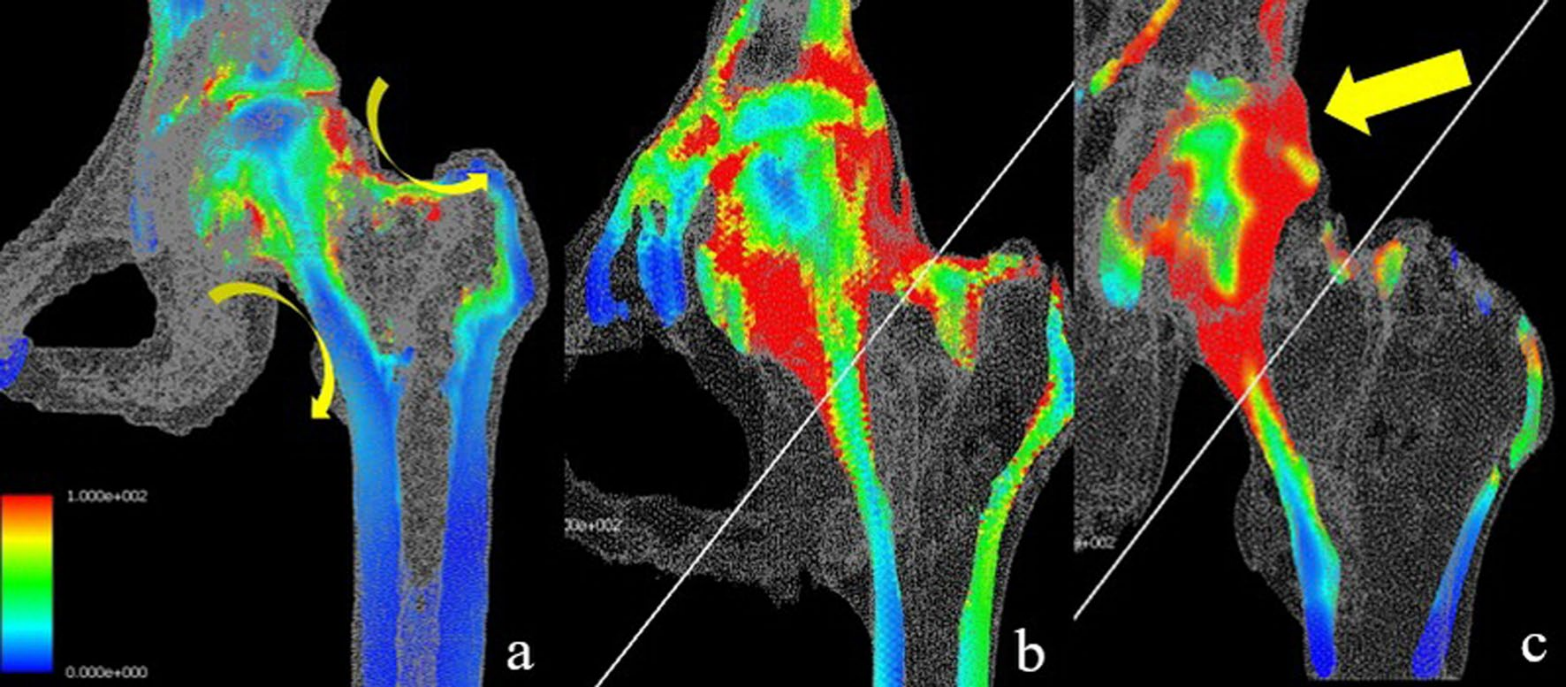
Representative final results of FEM analysis. Stress dispersion to the medial and lateral cortices (curved arrows) was significantly dominant (81.2%) in non-collapsed group (**a**). Intermediate results (**b**) were regarded as stress dispersion in the analysis. On the contrary, stress concentration to the lateral pillar (straight arrow) which proceeds to the primary compression trabeculae was markedly observed (87.5%) in the collapsed group (**c**)

Primary outcome was set as equivalents for yield stress (%) which signifies the compression force applied around the hip joint. The ultimate outcome of the FEM was provided in the visual form of figures which quantitatively display the coronal cross sections of the joint, hence intuitively showing either stress concentration or stress dispersion is being applied to the proximal femur. The results were classified as stress dispersion (Fig. 3a, b) when no focal concentration of stress was observed, and as stress concentration (Fig. 3c) when convergence of load transfer to the lateral pillar was clearly observed.

### Statistical analysis

Variables of age, sex, and BMI were used for propensity score analysis between collapsed and non-collapsed groups. Paired t test was used to confirm match design. Mann–Whitney tests were used for analysis of numerical variables including age, BMI, and necrotic size. In addition, Fisher’s exact tests were used to compare gender, location of necrosis, and the final results of stress distribution between the two groups. All statistical analyses were performed through Statistical Package for the Social Sciences (Version 20.0; SPSS, Chicago, Illinois, USA). For propensity score matching, PS Matching R plugin (version 3.0) on R software was used (Version 2.12.0, R Development Core Team, Vienna, Austria). Cutoff p-value of < 0.05 was used to determine statistically significant results.

## Results

Demographics of age (49.4 ± 14.2 vs. 48.7 ± 13.5, *p* = 0.958) and gender (male to female, 9:7 vs. 9:7, *p* = 1.000) exhibited no significant difference between the two groups, reassuring propensity-scored matching. In addition, BMI also had no significant difference (23.3 ± 4.7 vs. 25.1 ± 6.2, *p* = 0.944) (Table 1). Locations of the necrosis were all in the lateral trisection for group A, while for the group B they were distributed along the central or medial (*n* = 8) and lateral (*n* = 8) trisections (*p* = 0.015). Size of the bone affected by necrosis was larger in group B (46.8 ± 20.3% vs. 64.1 ± 33.6%, *p* = 0.015) (Table 2). Upon reassessment at over one-year follow up, patients in group A had resulting ARCO stage of 2B (*n* = 2), 2C (*n* = 10), 3B (*n* = 1), and 3C (*n* = 3), whereas all patients in group B proceeded to group 3C (*n* = 16) who experienced progression of a collapse.

**Table 1:** Demographics of 32 hips in 32 distinct patients with ONFH

Propensity-score parameters	End results after follow-up		*p*-value
	Non-collapsed	Collapsed	
Age (years)	49.4 ± 14.2 (21–75)	48.7 ± 13.5 (26–79)	0.958
Sex (male: female)	9: 7	9: 7	1.000
BMI (kg/m^2^)	23.3 ± 4.7	25.1 ± 6.2	0.944

**Table 2:** Initial characteristics of osteonecrosis and FEM analysis results of stress distribution (*p < 0.05)

Initial characteristics of osteonecrosis	End results after follow-up		*p *value
	Non-collapsed	Collapsed	
Location (central or medial: lateral)	8: 8	0: 16	0.015*
Size of necrosis (%)	46.9 ± 20.3 (16.7–85.8)	64.1 ± 33.6 (64.1–92.2)	0.015*
Stress concentration (yes: no)	3: 13	14: 2	0.001*

For the end result, among group A, most cases exhibited stress dispersion (*n* = 10) which was achieved by distribution of forces diverging to medial and lateral pillars (Fig. 3a), while a portion (*n* = 3) had less discrete dispersal but still stress did not concentrate to the lateral pillar nor the primary compression trabeculae as shown in Fig. 3b. Few showed stress concentration through the lateral pillar (*n* = 3). Overall, stress dispersion mainly consisted of group A (*n* = 13 (81.2%)).

On the other hand, in group B, stress concentration was mostly observed (*n* = 14 (87.5%)) where the stress is mainly delivered through the hip joint in a penetrating fashion, especially focusing on the lateral pillar of the femoral head, which then vertically converges along the primary compression trabecula of femoral neck as shown in Fig. 3c. Minority of group B exhibited stress dispersal (*n* = 2), which still showed collapse progression and required total hip arthroplasty.

Altogether, stress dispersal was mostly observed in the non-collapsed group whereas stress concentration was dominantly observed in the collapsed group (*p* = 0.001) (Table 2).

## Discussion

The prediction of collapse progression has been the key question in treating ONFH patients. Evolvement of classification systems and techniques in interpreting imaging studies were directed to elucidate such inquiry. Thus, indications of each treatment options including conservative care, joint preserving techniques, or arthroplasties have been widely studied, with a goal of basic consensus to preserve one’s natural hip joint as long as possible. In a recent study based on U.S. nationwide database, Sodhi et al*.* reported rates of arthroplasty (94.03%) were far greater than those of other procedures including osteotomy, partial arthroplasty, core decompression, and bone graft [15]. However, in young population, joint preserving techniques should be considered in prior to arthroplasties due to the latter’s higher complication rate, invasiveness, and implant life span which could lead to revision surgery [4, 15, 16].

In particular, biomechanical significance of the lateral pillar had been suggested in preventing collapse [9, 10, 17], but to date, cohort studies of which were scarcely reported [11]. Thus, we designed a study of propensity-score matched cohorts to further minimize selection bias prone in retrospective data analysis. Additionally, FEM analysis was selected to investigate the fact that not all cases of osteonecrosis placed in the lateral trisection undergo collapse, presumably due to the form of stress transmission rather than its sole mechanical structures of the osteonecrosis. Hence, emphasis of this study is not based on the absolute force on the lateral pillar but the presence of stress concentration on the critical areas determines collapse progression. Also, in this setting, it looks impracticable to set an absolute cutoff value in predicting collapse, because it may vary under various local and global conditions among different individuals’ hip joints.

For demographics, age (*p* = 0.958), sex (*p* = 1.000), and as well as BMI (*p* = 0.944) between the two groups had no significant difference as propensity scoring was estimated for age, sex, and BMI to minimize confounders. Non-collapsed hips for osteonecrosis over ARCO stage 2B are relatively rare compared to collapsed hips, thus the number of patients in the non-collapsed group was first swet then it was propensity-score matched to the cohort of collapsed patients. For pre-analytic comparison of characteristics of necrotic areas, location was classified as ‘lateral’ or ‘non-lateral’, where the latter includes centrally or medially located lesion based on the significance of the lateral pillar. All necrotic lesions in the collapsed group had pre-collapse lesions in the lateral trisection (*n* = 16), while lesions in the non-collapsed group were located half in the lateral (*n* = 8) and the other half in the non-lateral (*n* = 8) trisections. As a result, location (*p* = 0.015) and size (*p* = 0.015) had significant difference, which follows the current understandings of intrinsic risk factors of necrosis progression.

Upon the hypothesis of stress concentration on the lateral pillar might accelerate collapse, FEM analysis was conducted. To reflect extreme forces that can be applied to the hip joint, arbitrary stress load of 4500 N was set considering that up to 870% of body weight can be applied when stumbling in a 53 kg individual [18]. Internal mesh generation with 1-mm sized tetrahedral element and its subsequent analysis offers considerably fine FEM generation for analysis, but it requires high performance hardware for the software operation. To our knowledge, there had been no reports of FEM analysis for ONFH which uniformly used 1-mm tetrahedral elements [8–10].

As a result, stress concentration was focused on the lateral pillar in 87.5% of hips in the collapsed group while stress dispersion through the pillars were observed in 81.2% of the hips in the non-collapsed group (*p* = 0.001). Additionally, when stress concentration to the lateral pillar was present, distal force transmission through the primary compression trabeculae were always coupled. Thus, stress concentration converging to the vertical axis would predict near-future collapse with high probability, whereas stress dispersion through medial and lateral cortices of proximal femur is crucial in maintaining support of the anatomical structure. Therefore, the importance of structural support is emphasized not only at the lateral pillar, but inevitably also the primary compression trabeculae owing to its extended transmission of the yield stress distally.

Limitations of this study include small sample size of 16 patients per cohort, hence propensity-score matching was used to reinforce clinical significance given the small size in this retrospective case cohort study. Second, this study lacks quantitative analysis via such as von Mises stress [9, 11], stress index [7], or value of equivalent stress [8]. But as stated above, emphasis of this study is put on the qualitative cognition on the stress distribution rather than suggesting quantified cutoff values. In turn, proving effects of reinforced support to the lateral pillars using buttresses such as with fibular strut grafts or tantalum rod implantation would be analyzed through FEM in the future studies.

In conclusion, FEM analyses of followed-up ONFH suggest stress concentration to the lateral pillar and the primary compression trabeculae predicts collapse with high probability. Graphical output as an end result in pre-collapse ONFH provides a simple and intuitive, yet valuable information to aid surgeons in treatment selection. Moreover, FEM generation is achieved easily using ordinary CT data of a patient, depicting stress distribution which can be recognized at a glance. Therefore, especially for young patients, holding out the lateral pillar and the primary compression trabeculae through joint preserving procedures might be the key in preventing further collapse of the femoral head.

## Data Availability

The datasets used and analyzed during the current study are available from the corresponding author on reasonable request.

## Abbreviations

ARCO: Association Research Circulation Osseous
BMI: Body mass index
CT: Computed tomography
DICOM: Digital imaging and communication in medicine
FEM: Finite element model
MRI: Magnetic resonance imaging
N: Newtons
ONFH: Osteonecrosis of the femoral head
SPSS: Statistical Package for the Social Sciences
